# FK506 increases susceptibility to musculoskeletal infection in a rodent model

**DOI:** 10.1186/s12891-022-05667-1

**Published:** 2022-07-27

**Authors:** Stefanie M. Shiels, Preeti J. Muire, Joseph C. Wenke

**Affiliations:** Combat Wound Care, U.S. Army Institute of Surgical Research, JBSA-Fort Sam Houston, TX 78234 USA

**Keywords:** Immunosuppression, *Staphylococcus aureus*, Th1

## Abstract

**Background:**

Delayed fracture healing caused by soft tissue loss can be resolved by the administration of a Th1 immunosuppressant, such as FK506. Additionally, open fractures are at high risk for infection. We hypothesized that the inclusion of an immunosuppressant to a subject at risk for a musculoskeletal infection will increase the likelihood of infection.

**Methods:**

A rat model of musculoskeletal infection was used. Sprague Dawley rats received a stabilized femur defect and were inoculated with 10^4^ CFU *Staphylococcus aureus* via a collagen matrix. Six hours after inoculation, the wounds were debrided of collagen and devitalized tissue and irrigated with sterile saline. The animals were randomized into two groups: carrier control and FK506, which were administered daily for 14 days and were euthanized and the tissues harvested to measure local bioburden.

**Results:**

The dosing regimen of FK506 that restored bone healing increased the bioburden in the bone and on the fixation implant compared to the carrier control animals. As expected, the administration of FK506 decreased circulating white blood cells, lymphocytes, neutrophils, and monocytes. Additionally, the red blood cell count, hematocrit, and body weight were lower in those animals that received FK506 compared to carrier control.

**Conclusions:**

FK506 administration decreased the systemic immune cell counts and increased the bacterial bioburden within a model of musculoskeletal infection. Collectively, these outcomes could be attributed to the overall T cell suppression by FK506 and the altered antimicrobial activity of innate cells, thereby allowing *S. aureus* to thrive and subsequently leading to infection of severe, musculoskeletal injuries. These observations reveal the crucial continued investigation for the clinical use of FK506, and other immunosuppressant compounds, in trauma patients who are at increased risk of developing infections.

## Background

Severe open fractures, particularly with substantial soft tissue damage, have increased rates of complications such as nonunion and infection [1–3]. When primary closure is not possible, both rotational and free muscle flaps are often used to provide soft tissue coverage [4]. These surgical techniques are known to improve fracture repair and decrease infection rates but come with several disadvantages. First, cost of medical care can limit the number of time consuming free tissue transfers performed [5]. In addition to cost, free tissue transfers are more technically demanding, requiring surgeons skilled in microvascular surgery and tissue transfer techniques. The experience and training necessary for surgical proficiency is not available at all institutions and limits the number of successful free transfers [6]. Finally, free tissue transfers can result in donor site morbidity and scarring, reducing the overall quality of life. These limitations warrant a non-surgical approach to manage large open fractures while restoring fracture healing.

Although flaps are generally understood to improve open fracture healing by improving blood flow to the mangled tissues and by providing beneficial factors to the fracture area, recent work has demonstrated that concomitant lack of skeletal muscle coverage alters the local immune responses which subsequently delays fracture healing [7, 8]. More specifically, the lack of soft tissue coverage triggers increased local infiltration of T helper (CD4+) and cytotoxic T (CD8+) lymphocytes [7]. The influx of these T lymphocytes is believed to be partially responsible for the lack of boney union associated with the absence of skeletal muscle coverage, but also provides potential targets for immunosuppressive therapies [9]. FK506, a calcineurin inhibitor initially approved for use with solid organ transplantation, primarily functions by impairing T lymphocyte proliferation by inhibiting interleukin 2 (IL-2) production by CD4+ T lymphocytes [10]. Our group identified FK506’s potential to restore normal bone healing in the presence of concomitant muscle loss by moderating local T lymphocytes [11]. In short, our group developed a rat model which demonstrates the impairment of fracture healing when the fracture is accompanied by adjacent soft-tissue loss. The administration of daily FK506 within this model reduced the number of CD4+ T lymphocytes within the injured skeletal muscle and CD4+ and CD8+ T lymphocytes in the bone callus resulting in restoration of normal fracture healing. This provides evidence that pharmacological approaches that reduce the adaptive immune response may be promising therapies to restore endogenous fracture healing associated with severe muscle trauma.

These large musculoskeletal injuries also have high infection rates, particularly from gram-positive organisms, with *Staphylococcus aureus* being the most common [12, 13]. Under normal circumstances, *S. aureus* activates both innate and adaptive immune systems of the host via the activation of specialized pattern recognition receptors such as toll-like receptors (TLRs) on innate immune cells [14]. In fact, *S. aureus* enterotoxins can cause an activation of 2–20% of all T lymphocytes compared to other antigens which activate approximately 1 in 10,000 T lymphocytes [15]. Additionally, osteoblasts, which internalize the *S. aureus*, activate innate and adaptive immune responses by releasing series of cytokines and chemokines [16]. These immune systems work simultaneously to mitigate infection. Considering FK506 alters the activation of T lymphocytes, a major cellular component of the adaptive response, we postulate that the inclusion of this immunosuppressant may increase the likelihood of infection. Most importantly, to better understand the potential of immunosuppression as an alternative for muscle flaps, we investigated if the FK506 regimen that restored fracture healing increases *S. aureus* infection in a rodent model of open fracture.

## Methods

Animal research was conducted in compliance with the Animal Welfare Act, the implementing Animal Welfare Regulations, and the principles of the Guide for the Care and Use of Laboratory Animals. The Institutional Animal Care and Use Committee of the US Army Institute of Surgical Research approved all research conducted in this study. The facility where this research was conducted is fully accredited by The Association for Assessment and Accreditation of Laboratory Animal Care (AAALAC).

A musculoskeletal wound model was used to evaluate the risk of implant-associated infection with the use of FK506 [17–20]. Anesthetized Sprague-Dawley rats (*N* = 22) were premedicated with slow-release buprenorphine and anesthetized with gaseous isoflurane. The right hind limb was shaved and prepared for surgery by alternating alcohol and betadine scrubbing. For each animal, the femur was exposed with combination of sharp and blunt dissection and a poly-acetal plate was affixed to the anterior surface with six stainless steel threaded Kirschner wires. A 6 mm section of bone was removed by reciprocating saw under copious saline. Collagen was presoaked with 10^4^ colony forming units (CFU) of *Staphylococcus aureus* (Xen36, Perkin Elmer), a dose expected to cause infection in approximately 50% of the animals, and placed within the defect space [21]. The tissues were closed with suture and skin clips and the animals recovered. Six hours following initial surgery, a general clinical goal to initiate surgical care of an open fracture, the animal was again anesthetized, the wound re-opened, debrided of collagen and devitalized tissue, and irrigated with 60 cc normal saline (I&D) [20, 22, 23]. The wound was reclosed with suture and skin clips, recovered, and randomized into one of two groups: vehicle control (60% saline, 40% ethanol, *n* = 11) or FK506 (1 mg/kg at 5 mg/ml in vehicle, n = 11), which is the same dose that restored bone healing [11]. The vehicle of 60% saline and 40% ethanol was chosen due to FK506’s solubility characteristics. Starting immediately following I&D, animals were administered either vehicle control or FK506 intraperitoneal once daily for 14 days. Additionally, each animal received 5 mg/kg cefazolin SC BID for 72 hours following I&D [21]. The animals were monitored closely for signs of distress, including loss of weight, appetite, and mobility. Fourteen days after surgery, the animals were anesthetized, blood collected from cardiac puncture for complete blood count (CBC; Advia 2120i, Siemens, Malvern, PA), euthanized with overdose pentobarbital, and hind limbs harvested for bacterial bioburden. Bones and implants were aseptically collected and separated. Bones were snap frozen, crushed to a fine powder, resuspended in saline and vortexed for 10 minutes. Implants were sonicated in saline for 10 minutes to remove surface bacteria. Serial dilutions of bone and implants homogenates were plated onto blood agar and incubated overnight at 37 °C. CFU were normalized to sample weight.

### Statistical analysis

Normally distributed weight and CBC data are described as mean ± standard error of the mean. Non-normally distributed CFU data are described as median ± interquartile range (IQR). Data were analyzed using t-test or one-sided Mann-Whitney test where appropriate. Infection rate was defined as samples of greater than 10^3^CFU/g compared to total samples in the group and differences determined with one-sided Fisher’s exact test.

## Results

**Fig. 1:**
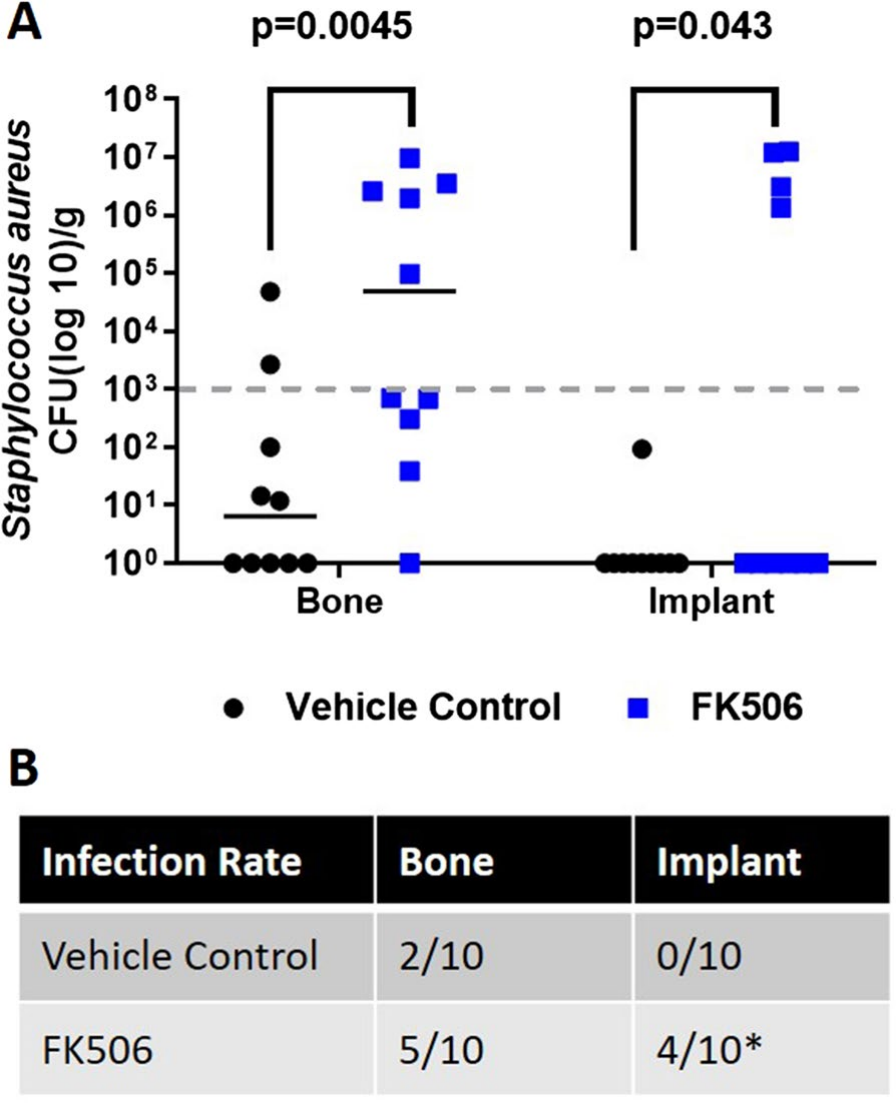
**A** *Staphylococcus aureus* (log_10_ CFU) recovered from bone tissue and implant surface. Normalized bioburden (# CFU/g sample), represented as individual values, within both the bone and implant of the FK506 group, are statistically greater than the bacteria recovered from the vehicle control animals, *p* = 0.0045 and *p* = 0.043, respectively. **B** The infection rate within the bone tissue and on implants of animals that received the vehicle control or FK506 therapy. There are no differences in infection rate between groups in the bone samples (*p* = 0.17). There are differences in infection rate on the implant between the vehicle control and the FK506 treated animals (*,*p* < 0.043)

FK506 significantly increased the bacterial bioburden within the bone. The median CFU in the bone for the vehicle control was 6.5 × 10^0^ (IQR 1–7.5 × 10^2^), whereas the median in the bone that received once daily FK506 was 4.9 × 10^4^ (IQR 1–2.9 × 10^6^) (*p* = 0.0045) (Fig. 1A). As with the bone tissue, there were more bacteria on the implants of animals treated with FK506 (*p* = 0.043) (Fig. 1A). There was not a statistical difference in infection rate within the bone (*p* = 0.17); two animals were infected in the control and five in the FK506 group (Fig. 1B). There was, however, a significant differences in the infection rate on the implant (*p* = 0.043) between groups, with zero implants infected in the control group and four in the FK506 group (Fig. 1B).

The animals in the FK506 group lost significantly more weight (12.49 ± 1.04%) compared to the vehicle control group (3.11 ± 1.21%) (*p* < 0.0001). FK506 also significantly decrease in CBC components such as white blood cells, monocytes, neutrophils, lymphocytes, RBCs and hematocrit (Table 1). Two animals, one from each group, expired prior to the conclusion of the study and were excluded from the data. Of these, one animal was euthanized due to failure to thrive and the other was found dead in its cage, both within 3 days of initial surgery.

**Table 1:** Systemic complete blood count (CBC) at the point of euthanasia

	WBC (× 10^3^)	RBC (× 10^6^)	HCT (%)	Neut (× 10^3^)	Lymph (× 10^3^)	Mono (× 10^3^)
Vehicle Control	11.95 ± 0.96	8.19 ± 0.16	45.84 ± 0.74	1.71 ± 0.37	9.27 ± 0.80	0.60 ± 0.22
FK506	5.88 ± 0.62^a^	7.62 ± 0.20^c^	40.94 ± 0.81^b^	0.85 ± 0.16^c^	4.71 ± 0.49^a^	0.18 ± 0.03^b^

*WBC* white blood cell, *RBC* red blood cell, *HCT* hematocrit, *Neut* neutrophils, *Lymph* lymphocytes, *Mono* monocytes

^a^*p* < 0.0001, ^b^*p* < 0.001, ^c^*p* < 0.05

## Discussion

This study provides evidence that the use of the dosing regimen of the immunosuppressant, FK506, that restores bone healing of segmental defects accompanied by severe soft tissue damage, increases the susceptibility of the wound to localized infection from *S. aureus*. When given daily, in conjunction with a systemic broad-spectrum antibiotic, those animals that received FK506 had significantly more localized *S. aureus* in both the bone and on the implant, greater weight loss, and altered leukocytes compared to those animals that received the vehicle only control. With this, it is crucial to consider this risk and take increased precautions when utilizing FK506 for fracture repair.

A warning of increased infection risk accompanies a number of therapies and conditions. An entire class of drugs are used to suppress the adaptive immune response to improve graft and organ integration and to modulate over-active autoimmune disorders such as Lupus [24]. Similar to FK506 and other calcineurin inhibitors, anti-proliferatives, such as azathioprine, and mTOR inhibitors, such as rapamycin, decrease T cell production [25]. Inhibition of the mTOR pathway is associated with an increased incidence of infection, resulting in an increased number of subjects receiving antibiotics compared to the control subjects in ten Phase I Clinical Trials [26]. Moreover, patients infected with human immunodeficiency virus (HIV) become CD4+ T cell deficient over time [27], thereby making them prone to opportunistic bacterial and fungal infections [28]. Abalo et al. identified a correlation between HIV induced low CD4+ T cell number and surgical site infection risk following orthopaedic trauma surgery [29]. A study demonstrated in a mouse model of *S. aureus* infection that the reduction of CD4+ T helper type (Th1) cells, part of the adaptive immune response that activates macrophage and phagocytosis, permits *S. aureus* infection to thrive [30]. More importantly, the memory Th1 cells are critical to *S. aureus* infection [31]. With this, it is understandable to assume that the use of FK506, which attenuates T cell proliferation and would likely increase the risk of infection of an open fracture. Open fractures are at a high risk for infection with rates in Gustilo-Anderson type IIIB open tibial fractures upward toward 43% [2].

**Fig. 2:**
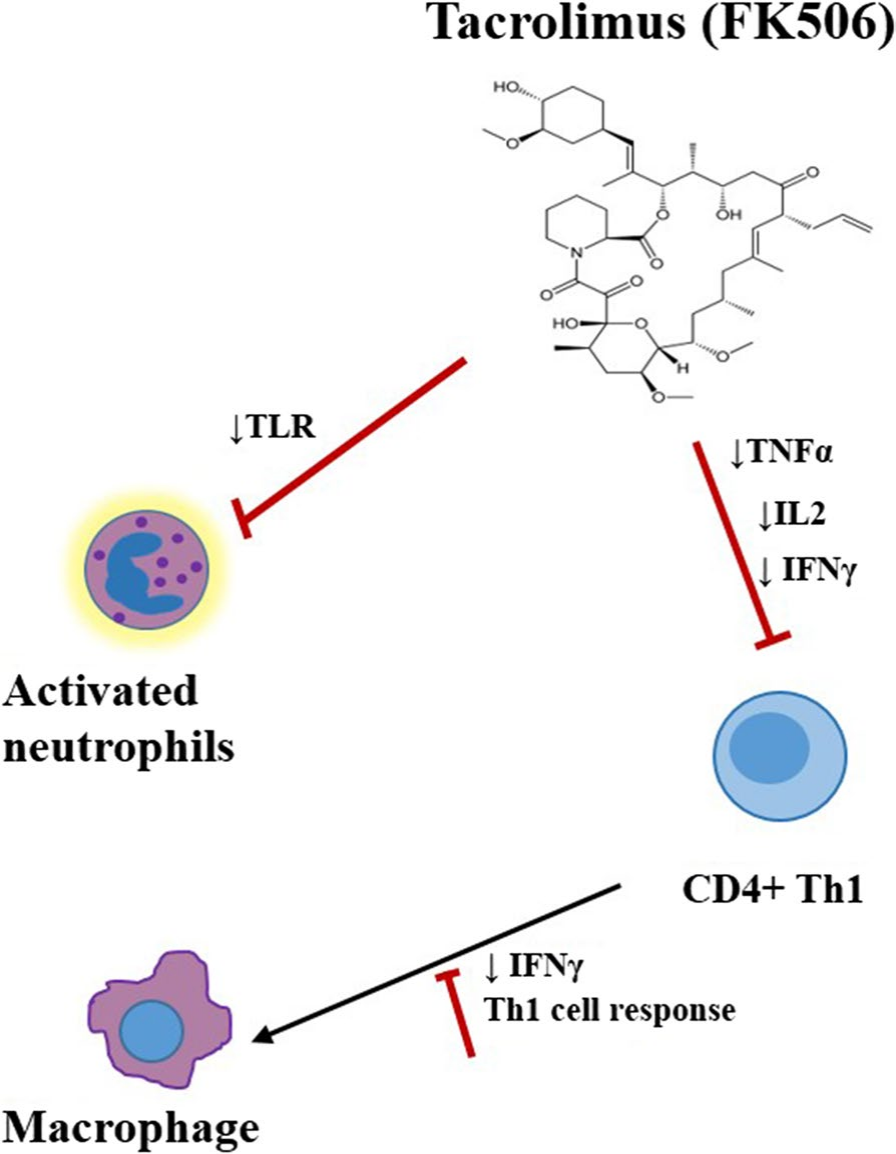
The effect of FK506 on immune function. The primary mechanism of FK506 is to inhibit the IL-2 production that is required for CD4+ T lymphocyte activation and function. In such a case, TNF-α and IFNγ expression are reduced. Lack of CD4+ T lymphocyte activation leads to decreased phagocytic activity of macrophages. Additionally, FK506 also effects neutrophil activation by attenuating Toll-like receptors (TLR) responses required for the innate immune responses

FK506 suppresses T lymphocyte responses by first forming a complex with the intracellular FK binding protein and then inhibiting the binding of calcineurin to calmodulin [10]. Inhibition of calcineurin inactivates nuclear factor activation of activated T-cells (NF-AT), which is essential for interleukin 2 (IL-2) gene transcription. A break in the normal circuit leads to disruption of the IL-2 dependent Th1 cell activation and proliferation (Fig. 2). Macrophage activation is a key effector of the Th1 cell activation [32]. By inhibiting Th1 cell activation, Th1 specific IFNγ and TNFα cytokine production is also inhibited [33, 34]. Subsequently, it is possible that the resident macrophages at the wound site lack IFNγ and TNFα mediated activation signals and remain in a dormant state, thereby limiting their ability to kill bacteria. Additionally, calcineurin inhibitors also have inhibitory effects on macrophages and monocytes by reducing TLR activation, preventing cytokine secretion, and inhibiting effective antigen presentation of antigen presenting cells (APCs) to further activate Th1 cells [35]. Understandably, it is assumed that an alteration in Th1 cells activation by FK506 could cause an increased risk for wound infection but this has never been determined in a model of musculoskeletal infection.

**Fig. 3:**
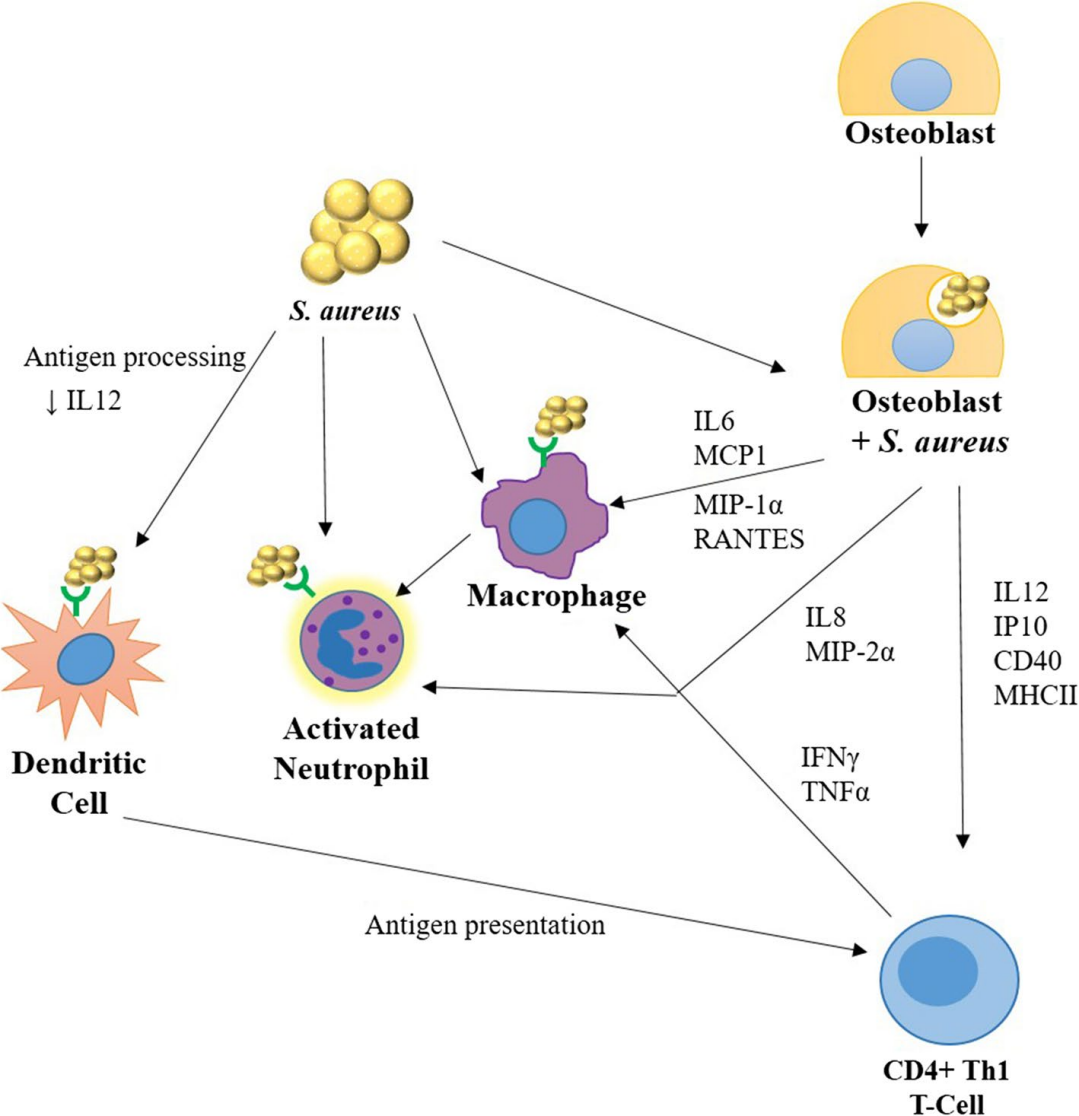
Activation of the immune system by *S. aureus*. *S. aureus* pathogen associated molecular pathogens (PAMPS) are recognized by the TLRs of macrophages, neutrophils, and DCs to initiate the innate immune response. Additionally, bacteria stimulate osteoblasts to express a number of cytokines, such as interleukins − 6, − 8, and − 12 (IL6, IL8, and IL12), chemokines, such as monocyte chemoattractant protein 1 (MCP1), macrophage inflammatory proteins 1α and 2α (MIP-1α and MIP-2α), RANTES, and interferon gamma induced protein 10 (IP-10), and proteins CD40 and MHCII. Together these activate both innate immunity, through macrophage activation, and adaptive immunity, through CD4+ Th1 T-cell activation

*S. aureus* activates both the innate and adaptive immune responses, particularly macrophage recruitment and activation through their pathogen associated molecular patterns (PAMPs) [14]. Directly, dendritic cells, resident macrophages and recruited neutrophils are activated by the recognition of bacterial PAMPs via their pattern recognition receptors such as TLRs (Fig. 3). Activation leads to *S. aureus* phagocytosis, degranulation and entrapment in neutrophil secreted traps. Indirectly, *S. aureus* induces osteoblasts, either by contact or internalization, to secrete chemokines and cytokines, such as IL-6, MCP-1, MIP-1α, Regulated upon Activation, Normal T Cell Expressed and Presumably Secreted (RANTES), IL-8, and MIP-2α as well as surface markers such as CD40, and MHC II, which then recruit and activate both the innate and adaptive immune responses (Fig. 3) [16]. Of note, release of IL-12 and IP-10, two potent recruiters of activated Th1 cells, is increased (ref). Th1 cells function to control and coordinate host defense against pathogens, by releasing a range of other cytokines, chemokines, and surface molecules that mediate killing of chronically infected senescent macrophages in bone marrow and recruit circulating monocyte derived macrophages to the site of the infection. Without the activation by Th1 cytokines TNFα and IFNγ, macrophages are present but may be unresponsive to invading *S. aureus* [36, 37]. Macrophages require signaling to activate and maintain their activation state. These signals include the potent macrophage activating IFNγ produced by Th1 cells and a membrane bound signal needed to sensitize the macrophage to the IFNγ in order to remain activated. These sensitization signals can be provided by Th1 cells as the expression of CD40 ligand that binds to the CD40 receptor on macrophages to further stimulate macrophage phagocytic activity.

There are several limitations to this study. Firstly, by using only gram positive *S. aureus*, the conclusions are limited to a single infection causing pathogen. *S. aureus* is, however, the most prominent musculoskeletal infection causing organism and therefore is a fair representative. Additionally, we understand that there are other agents that suppress CD4+ activation that may similarly restore bone healing, such as cyclosporine. Although FK506 was the only drug investigated, it is also the only immunosuppressant that has been explored for fracture healing of severe open fractures with combined muscle trauma. Thirdly, other than complete blood counts, further immune cell profiling was not performed. This being said, the CBC data provides information to indicate the immunosuppressant activity of FK506 within the total peripheral blood lymphocytes..

## Conclusion

This study provides initial data regarding the potential infectious risk when using FK506 to restore fracture healing of severe musculoskeletal injuries. Although it has been shown to normalize the dysregulated CD4+ Th1 cells to restore natural fracture healing, FK506 also increases the susceptibility to acquire a *S. aureus* infection. While there are infectious complications, the use of an immunosuppressant may be a potential therapeutic for poor fracture healing caused by immune dysregulation, especially those that target the specific pathway.

## Data Availability

All data generated or analysed during this study are included in this published article.

## Abbreviations

AAALAC: The Association for Assessment and Accreditation of Laboratory Animal Care
APC: Antigen Presenting Cells
BID: twice-daily administration
CBC: Complete Blood Count
CD4+: T-helper cells
CD8+: Cytotoxic T cells+
CD40: CD40 molecule, a protein coding gene
CFU: Colony Forming Units
HIV: Human Immunodeficiency Virus
I&D: Irrigation & Debridement
IFNγ: Interferon γ
IL-2: Interleukin-2
IL-6: Interleukin-6
IL-8: Interleukin-8
IQR: Interquartile Range
MCP-1: Monocyte Chemoattractant Protein-1
MHCII: Major Histocompatibility Complex II
MIP-1α: Macrophage Inflammatory Protein-1α
MIP-2α: Macrophage Inflammatory Protein-2α
NF-AT: Nuclear factor of activated T-cells
PAMPs: Pathogen-Associated Molecular Patterns
RANTES: Regulated upon Activation, Normal T Cell Expressed and Presumably Secreted
RBC: Red Blood Cells
*S. aureus*: *Staphylococcus aureus*
SC: Subcutaneous
TFNα: Tumor Necrosis Factor α
Th1: T helper type 1
TLR: Toll-like Receptors
